# Enhanced antisaccade abilities in children with Tourette syndrome: the Gap-effect Reversal

**DOI:** 10.3389/fnhum.2013.00768

**Published:** 2013-11-13

**Authors:** Diana J. Tajik-Parvinchi, Paul Sandor

**Affiliations:** ^1^Department of Psychology, Centre for Vision Research, York UniversityToronto, ON, Canada; ^2^Tourette Syndrome Neurodevelopmental Clinic and Toronto Western Research Institute, University Health NetworkToronto, ON, Canada; ^3^Department of Psychiatry, University of TorontoToronto, ON, Canada; ^4^Youthdale Treatment Centers, YDL InstituteToronto, ON, Canada

**Keywords:** Tourette syndrome, saccades, children, gap effect, antisaccade, cognition

## Abstract

Tourette Syndrome (TS) is a childhood onset disorder of motor and vocal tics. The neural networks underlying TS overlap with those of saccade eye movements. Thus, deviations on saccadic tasks can provide important information about psychopathology of TS. Tourette syndrome often coexists with Attention Deficit Hyperactivity Disorder (ADHD) and Obsessive Compulsive Disorder (OCD). Hence, we manipulated various components of a saccade task to measure its effects on saccades of children with TS-only, TS+ADHD, TS+ADHD+OCD and healthy controls. Children looked toward (prosaccade) or in the opposite direction (antisaccade) of a peripheral target as soon as it appeared. The prosaccade and antisaccade tasks were presented in three conditions. In the Gap200 condition, the fixation dot disappeared 200 ms prior to the appearance of the peripheral target, In the Gap800 condition, the fixation dot disappeared 800 ms prior to the appearance of the peripheral target and in Overlap200 the fixation dot disappeared 200 ms after the appearance of the peripheral target. Fixation-offset manipulations had different effects on each group's antisaccades. The TS+ADHD+OCD group's antisaccade latencies and error rates remained relatively unchanged in the three conditions and displayed a pattern of eye movements that can be interpreted as enhanced. Alternatively, the TS+ADHD group displayed an overall pattern of longer saccadic latencies. Findings corroborate the hypothesis that the combination of tic disorder and ADHD results in unique behavioral profiles. It is plausible that a subgroup of children with TS develop an adaptive ability to control their tics which generalizes to enhanced volitional control of saccadic behavior as well. Supporting evidence and other findings are discussed.

## Introduction

Tourette Syndrome (TS) is a childhood onset disorder of motor control with the primary symptoms consisting of motor and vocal tics. A tic is a sudden and stereotypic motor movement or vocalization (DSM-IV-TR, 2004). Certain types of disorders such as Attention Deficit Hyperactivity Disorder (ADHD) and Obsessive Compulsive Disorder (OCD) are strongly linked to TS and may be an integral part of this disorder. ADHD is the most commonly encountered comorbid condition in TS, occurring in about 21–90% of this population (Burke and Reveley, 2002).

Deficits within the basal ganglia (BG), the limbic structures and areas of the frontal cortex to which the BG project through the thalamocortical pathways appear to be associated with TS (Segawa, 2003; Sweeney et al., 2004). Patients with TS show regional gray matter and/or white matter reductions in supplementary motor area, the premotor cortex, the sensorimotor cortex and the prefrontal cortex (Müller-Vahl et al., 2009). A decreased connectivity between the dorsolateral prefrontal cortex and the caudate nucleus has also been reported (Makki et al., 2009). Many of these areas have also been implicated in the generation of eye movements. The BG as well as Frontal Eye Field (FEF), dorsolateral prefrontal cortex and supplementary eye field have also been identified as part of the neural substrates responsible for control of eye movements (Sweeney et al., 1996; Connolly et al., 2005; Ford et al., 2005; Hutton and Ettinger, 2006; McDowell et al., 2008). Given this overlap of neural substrates between TS and eye movement control in addition to the motor disinhibition associated with TS, eye movement ability may be affected in these individuals and it may provide important information about the psychopathology of TS.

The present study focused on saccadic eye movements. Saccades are fast eye movements that redirect the gaze to different scenes and scan the visual environment (Leigh and Zee, 1999; Kowler, 2011). In this context, the ability to successfully collect visual information is highly dependent on saccadic reaction time, also referred to as saccade latency. Hence, factors that can alter saccadic latency are important and have attracted researchers' attention for many years.

A phenomenon that has been shown to consistently and predictably alter saccadic latency is the “Gap-effect.” Saslow (1967) observed this phenomenon in a prosaccade task. In a prosaccade task, commonly a fixation dot is presented in the center of a screen and participants fixate on it. Then, the fixation dot disappears and a peripheral target appears. Participants must generate a saccade to the peripheral target as soon it appears. Saslow observed that when the fixation dot disappeared shortly before the onset of the peripheral target (Gap condition), saccadic latency of adults decreased significantly relative to an Overlap condition, where the fixation dot remained visible at the Center of the screen at the onset of the peripheral target. Saslow termed this significant decrease in saccade latency in a Gap condition relative to an Overlap condition the “Gap-effect.”

Since Saslow's original discovery, many researchers have investigated this phenomenon and have consistently reported the same effect in humans and primates (Dorris and Munoz, 1995; Kopecz, 1995; Munoz et al., 1998; Klein and Foerster, 2001; Dick et al., 2005; Pratt et al., 2006). Several explanations have been put forward to account for this effect. One view attributes it to high-level cortical mechanisms controlling attention. According to this view, fixation removal disengages attention and when the target appears, attention is ready to be shifted toward it (Fischer and Weber, 1992; Rolfs and Vitu, 2007). Another view claims that when the fixation dot disappears, fixation activity in the superior colliculus (CS) decreases and the oculomotor system is prepared to generate a saccade (Munoz and Wurtz, 1993a,b; Dorris and Munoz, 1995; Everling et al., 1998). A general warning effect has also been suggested to be responsible for this phenomenon (Reuter-Lorenz et al., 1995).

The Gap-effect has also been observed in an antisaccade task (Hallett, 1978), where participants have to generate a saccade in the opposite direction of the peripheral target. This task involves: (1) The inhibition of a saccade toward the peripheral target (prosaccade); and (2) the volitional generation of a saccade in the opposite direction of the peripheral target (the antisaccade). Thus, an antisaccade involves inhibitory control (Roberts et al., 1994; Fukushima et al., 2000; Pratt et al., 2006) and demands working memory resources (Roberts et al., 1994; Malone and Iacono, 2002); whereas a prosaccade entails the generation of a saccade to a visual stimulus. Antisaccade eye movements take approximately 100–150 ms longer to initiate than prosaccades (Roberts et al., 1994; Malone and Iacono, 2002) and given the differences inherent in the two types of eye movements, data are commonly reported separately for each. In an antisaccade task, saccades made toward the target are referred to as direction errors, or antisaccade errors. Healthy adults' antisaccade latencies have been shown to decrease in a Gap condition relative to an Overlap condition (Gap-effect) (Fischer and Weber, 1992; Goldring and Fischer, 1997). However, antisaccade errors increase in a Gap condition relative to an Overlap condition.

Since Saslow's original study did not investigate Gap ranges beyond 350 ms, researchers have contemplated about possible effect of longer gaps on saccadic latency. There is limited research in healthy adults on this phenomenon. Fischer and Weber (1997) found a reversal of the Gap-effect with longer gap durations (600 ms). It has been suggested that an additional inhibitory mechanism may be responsible for the longer saccade latencies observed in the longer Gap conditions (Knox, 2009).

The Gap-effect phenomenon has also been observed in healthy children (Munoz et al., 1998; Klein and Foerster, 2001). To the best of our knowledge, there have not been any studies investigating the effect of longer gap durations in healthy children or children with TS. Presently, we only have a partial knowledge of the effect of fixation manipulations on saccadic performance of healthy children and children with TS. Moreover, we do not know the effect of fixation manipulations on saccades of children with TS+comorbid conditions such as TS+ADHD or TS+ADHD+OCD. The aims of the present study were to:

1. Compare healthy children's saccadic latency and error rates in a long Gap condition relative to an Overlap and to a short Gap condition; We selected the short Gap condition as the baseline condition since the definition of Gap-effect consist of a latency difference between a short Gap (200–250 ms) and an overlap condition. In the present study we wished to examine whether the Gap-effect would still emerges if the duration of the Gap increases.
2. Compare the same parameters as in (1) in sub-groups of children with TS, divided according to comorbidity status, relative to one another and relative to healthy children.

Given that children's prosaccade abilities are similar to those of adults (Munoz et al., 1998; Fukushima et al., 2000; Klein and Foerster, 2001; Eenshuistra et al., 2007), we expected to observe the “reversal of Gap-effect” in prosaccades of our healthy children's group. In other words, we anticipated longer prosaccade latencies in the long Gap condition relative to those in the short Gap condition. However, since children display higher rates of antisaccade errors relative to adults (Fischer et al., 1997; Munoz et al., 1998; Fukushima et al., 2000; Klein and Foerster, 2001; Eenshuistra et al., 2007), we anticipated the rate of direction errors not to decrease in the long Gap condition. In other words, we expected children's error rates to remain similar in the two Gap conditions, in contrast to that observed in healthy adults.

## Materials and methods

### Participants

Ten healthy children 10–16 years of age (*M* = 13, *SD* = 2.25) and 22 children with TS 8–16 years of age (*M* = 12.5, *SD* = 2.4) participated in this study. The children with TS were separated into three subgroups: TS-only (*n* = 6; Range: 97–151 months; Mean = 132 months, *SD* = 21.4), TS+ADHD (*n* = 9; Range: 105–195 months Mean = 145.4 months *SD* = 28.8), and TS+ADHD+OCD (*n* = 7; Range: 133–198 months Mean = 173.1 months, *SD* = 20.5). Many of the children with TS were receiving medication. These medications included Clonidine (2 TS-only; 4 TS+ADHD; 2 TS+ADHD+OCD), Risperidone (3 TS+ADHD; 2 TS+ADHD+OCD), Citalopram (TS+ADHD), Atomoxetine (1 TS+ADHD; 1 TS+ADHD+OCD), Fluvoxamine (1 TS+ADHD; 1 TS+ADHD+OCD), Quetiapine (1 TS+ADHD; 1 TS+ADHD+OCD), Bupropion (1 TS+ADHD; 3 TS+ADHD+OCD), Zoloft (1 TS+ADHD; 1 TS+ADHD+OCD), Dextroamphetamine (1 TS+ADHD+OCD), Olanzapine, Amantadine, Pantoprazole sodium, Salbutamol, Fluticasone (1 TS+ADHD+OCD), Amantadine (1 TS+ADHD+OCD). For a full demographic and medication information of each participant, please see Tajik-Parvinchi and Sandor (2011).

The diagnosis of TS was carried out according to DSM-III-R diagnostic criteria by an experienced neuropsychiatrist. The diagnosis of TS was carried out according to DSM-III-R diagnostic criteria by an experienced neuropsychiatrist. The DSM IIIR criteria were used because the DSM IV criteria include a tic-free period of no longer than 3 months—an unnecessary and impractical criterion for the diagnosis of TS. There is a literature supporting the use of the DSM IIIR over the DSM IV (Freeman et al., 1995; Spitzer and Wakefield, 1999). Spitzer and Wakefield (1999) discuss the issue in more general terms. In fact, this requirement will not be present in DSM V. In order to validate these diagnoses, an independent physician reviewed the patients' files and provided separate diagnoses for each of our TS participants. An inter-rater reliability analysis revealed high levels of agreement between the two physicians Kapp = 0.861 (*p* < 0.001). Information about symptoms associated with TS and OCD were collected using self-report and family report based on the tic inventory and ordinal severity scales of the Yale Global Tic Severity Scale (Leckman et al., 1989) and the symptom checklist and ordinal scales of the Yale-Brown Obsessive-compulsive scale (Goodman et al., 1989). The history of ADHD was elicited in a similar manner using a questionnaire based on parental reports (Conners et al., 1998). This information was reviewed and checked by an experienced neuropsychiatrist, who also performed a direct examination of each child included in this study before assigning the diagnoses.

The majority of the children within our TS groups were receiving medication: 2/6 in the TS-only group; 8/9 in the TS+ADHD group and 7/8 in the TS+ADHD+OCD group. Healthy children according to parental report had no neurological, vestibular, or ocular motor anomalies.

Tic severity was determined retrospectively, based on a review of clinical information by the same neuropsychiatrist. Life-time tic severity was assessed on a 3-point scale (1-mild, 2-moderate and 3-severe). The participants' ADHD symptom severities were assessed retrospectively using the Clinical Global Impression of ADHD symptoms (CGI) both for life time and at the time of data collection [for participants demographic and medication information see Tajik-Parvinchi and Sandor (2011)]. The rater was not aware of the oculomotor performances of the participants when making the ratings.

Data collection was not completed by 3/32 children: 1 child in the Control group (prosaccade Gap200; 83% of data available for this child), 2 children in the TS-only group (the prosaccade Gap200 condition, 83% of data available for each child) due to participant fatigue and technical difficulties with the system (i.e., the eye tracker crashing several times requiring recalibration). Please note that these children were able to complete majority of the tasks and the data from the completed tasks were included in the analyses.

### Ethics statement

Informed written consent was obtained from the parents of the children and informed written assent was attained from the children. This study was approved by the University Health Network Ethic's Board and complied with the tenets of the Declaration of Helsinki.

### Materials

Eye movements were measured with a video-based cornea/pupil tracking system (El-Mar Series 2020 Eye Tracker, Toronto, Canada) with a maximum resolution 0.1° of visual angle. This system is free of drifts and it has a linear range of greater than 30° horizontally and 25° vertically. Eye movements were sampled at 120 Hz. Participants were seated upright in a chair especially crafted to adjust to various heights, to ensure that all participants' eye levels were the same when viewing the stimuli presentations and pointed to the center of the screen. The head was stabilized with a chin-rest. The stimuli extended around 0.25° and consisted of a blue fixation dot and green/red dots representing the peripheral targets. The red dots were displayed for antisaccades and the green dots for prosaccades. The stimuli were back projected on a screen which was 200 cm from the seated participants. The eye tracker was calibrated for each participant prior to data collection. The eye movement data and the stimuli presentations were recorded by the same eye tracking system.

### Procedure

Children were presented with two tasks, the prosaccade and the antisaccade task. Each task was presented in 3 conditions. Thus, each participant had to complete 6 experimental conditions: prosaccade Gap200, prosaccade Gap800, prosaccade Overlap200, antisaccade Gap200, antisaccade Gap800, and antisaccade Overlap200. Each experimental condition consisted of 25 trials and was presented in a randomized order.

In the Gap200 condition, the fixation dot disappeared 200 ms prior to the onset of the peripheral stimulus; in the Gap800 condition, the fixation dot disappeared for 800 ms prior to the onset of the peripheral stimulus and in the Overlap200 condition, the fixation dot remained on for 200 ms at the onset of the peripheral stimulus. In the present study, the Gap200 condition served as a baseline condition relative to which saccadic alterations in Gap800 and Overlap200 were measured. Data on group differences in the Gap200 condition have been reported elsewhere (Tajik-Parvinchi and Sandor, 2011).

The peripheral stimuli were presented for 1000–1500 ms randomly at 5°, 10°, and 15° to the left and right of the central fixation dot, which had a duration of 1000 ms. Children were instructed prior to the presentation of each experimental condition and given an opportunity to ask questions. Also, the experimenter asked specific questions to determine whether the child had understood the task instructions such as “what do you do when you see a red dot to the left or right of the middle dot?” If the child answered the questions correctly and expressed understanding of the task instructions, the experiment proceeded. The task instructions in the prosaccade tasks were to look at the peripheral target as soon as it appeared. In the antisaccade tasks, they were instructed to look in the opposite direction of the stimulus but mirror distance from the fixation dot as soon as the target appeared.

### Data analysis

A custom designed software program, AnYZLL 3.3 displayed the eye movement data and the stimuli information. Eye movements with peak velocities greater than 50°/s were marked as saccades by AnYZll 3.3. The experimenter visually examined the marked saccades in order to confirm that the saccades were not blink artifacts. The saccade onset was calculated as the time at which saccadic velocity exceeded 10°/s. The first saccade generated within the 100–1000 ms of the stimuli onset was selected and further analyzed. Saccades with latencies outside of this time window were considered anticipatory saccades, secondary saccades or not in response to the stimulus. In an antisaccade condition, if the first saccade following the stimulus onset was in the same direction as the target, it was considered an antisaccade error. Similarly, in the prosaccade task, if the first saccade following stimulus onset was in the opposite direction of the stimulus, it was considered a prosaccade error. Only the correct saccades were included in the analyses of prosaccade and antisaccade latencies. Each subject's individual data was entered into a long SPSS data sheet where statistical analyses were carried out.

## Results

A Repeated measures analysis of variance (ANOVA) was carried. The dependent variables were: Antisaccade Latency, Prosaccade Latency and Antisaccade Error Rates. The factor Group (Control, TS-only, TS+ADHD, TS+ADHD+OCD) was entered as the between-subjects factor and Condition (Gap200, Gap800, Overlap200) was entered as the repeated measure factor, establishing a 4 × 3 mixed model. All pair-wise comparisons were corrected using the Bonferroni method.

### Prosaccade latency

#### Saccadic alteration across conditions

The ANOVA revealed a main effect of Condition *F*_(2, 51)_ = 96.225, *p* < 0.00, but there were no Group × Condition effect *F*_(6, 51)_ = 0.716, *p* = 0.638. However, given our priori hypothesis that the Gap-effect will weaken when Gap duration is increased, we followed up the ANOVA with Bonferroni corrected pairwise comparisons (Table 1). All groups displayed similar saccadic alterations across the three fixation offset conditions. All groups displayed the Gap-effect ranging in size from 27.8 to 57.12 ms for prosaccades. Overall, prosaccadic latencies increased significantly in the Gap800 condition relative to both Gap200 and the Overlap200 conditions. Hence, the Gap-effect Reversal was observed in all of the groups. For details see Table 1.

**Table 1:** **Average saccade latencies and error rates of each group across the three fixation offset conditions**.

	**Gap200**	***p*-Value, relative to X Condition**	**Gap800**	***p*-Value, relative to X Condition**	**Overlap200**	***p*-Value, relative to X condition**	**Gap-effect size**
**PROSACCADE LATENCY (ms)**							
Control (*n* = 10)	189.0^*^	*P* ≤ 0.001 Gap800;	274.2^*^	*P* < 0.001 Gap200;	221.0^*^	*P* < 0.001 Gap200;	32
		*P* < 0.001 Ovlerap200		*P* ≤ 0.001 Overlap200		*P* < 0.001 Gap800	
TS-Only (*n* = 6)	214.3^*^	*P* < 0.001 Gap800;	286.5^*^	*P* < 0.001 Gap200;	242.1^*^	*P* = 0.046 Gap200;	27.8
		*P* = 0.046 Overlap200		*P* < 0.001 Ovlerap200		*P* < 0.001 Gap800	
TS+ADHD (*n* = 9)	199.7^*^	*P* < 0.001 Gap800;	297.3^*^	*P* < 0.001 Gap200;	256.8^*^	*P* < 0.001 Gap200;	57.1
		*P* < 0.001 Overlap200		*P* < 0.001 Overlap200		*P* < 0.001 Gap800	
TS+ADHD+OCD (*n* = 7)	197.1^*^	*P* < 0.001 Gap800;	296.0^*^	*P* < 0.001 Gap200;	235.4^*^	*P* < 0.001 Gap200;	38.3
		*P* < 0.001 Overlap200		*P* < 0.001 Overlap200		*P* < 0.001 Gap800	
**ANTISACCADE LATENCY (ms)**							
Control (*n* = 10)	375.3		377.0		497.8^*^	*P* < 0.001 Gap200;	122.5
						*P* < 0.001 Gap800	
TS-Only (*n* = 6)	418.4		406.2		534.0^*^	*P* < 0.001 Gap200;	115.6
						*P* < 0.001 Gap800	
TS+ADHD (*n* = 9)	379.0^*^	*P* < 0.001 Gap800	452.9^*^		522.2^*^	*P* < 0.001 Gap200;	143.2
						*P* < 0.001 Gap800	
TS+ADHD+OCD (*n* = 7)	387.9		407.0		434.4^*^	*P* = 0.007 Gap200	46.5
**ANTISACCADE ERROR RATE (%)**							
Control (*n* = 10)	49.5^*^	*P* < 0.001 Gap800;	34.1		29.7		
		*P* < 0.001 Overlap200					
TS-Only (*n* = 6)	41.7		41.2		24^*^	*P* = 0.004 Gap200;	
						*P* = 0.005 Gap800	
TS+ADHD (*n* = 9)	48.2^*^	*P* = 0.01 Gap800	35.6		38.9		
TS+ADHD+OCD (*n* = 7)	28.9		30.3		32.2		

* Significant p-values for each pairwise comparison are provided.

#### Group differences

The ANOVA did not reveal an effect for Group *F*_(3, 28)_ = 0.863, *p* = 0.472.

### Antisaccade latency

#### Saccadic alteration across conditions

All of our groups displayed the Gap-effect for antisaccades ranging in size from 46.46 to 143.25 ms. The ANOVA revealed a main effect of Condition *F*_(2, 55)_ = 39.366, *p* < 0.001 and a Group × Condition effect, *F*_(6, 55)_ = 2.808, *p* = 0.02. However, the reversal of Gap-effect was not observed. All groups displayed shorter antisaccade latencies in the Gap800 condition relative to the Overlap200 condition (all *p*-values <0.01) except for one group, the TS+ADHD+OCD where this latency difference was not significant. Overall, it appears that increasing the Gap duration does not reverse the antisaccadic Gap-effect in any of our groups (Table 1).

#### Group differences

In contrast to our expectations, the TS+ADHD+OCD group displayed significantly shorter antisaccade latencies relative to the Control (*p* < 0.001), the TS+ADHD (*p* < 0.001) and the TS-only (*p* < 0.001) groups in the Overlap200 condition (Figure 2A) and also displayed significantly shorter antisaccade latencies relative to the TS + ADHD group in the Gap800 condition (*p* = 0.02). These findings indicate a faster reaction time in the TS+ADHD+OCD group. In contrast, the TS+ADHD group exhibited significantly longer antisaccade latencies (slower reaction times) in the Gap800 condition relative to the Control group (*p* < 0.001). In the Gap800 condition, the difference between the TS+ADHD and those of the TS-only group did not reach significance (*p* = 0.06).

### Antisaccade error rate

#### Error rate across conditions

The antisaccade error rate of each group altered in a unique way as a result of fixation offset condition (Figure 3A). The ANOVA revealed a main effect of Condition *F*_(2, 55)_ = 4.28, *p* = 0.02 and a Group × Condition interaction effect *F*_(6, 55)_ = 3.1, *p* = 0.01. The Control group had the highest rate of antisaccade errors in the Gap200 condition relative to the Gap800 (*p* < 0.001) and the Overlap200 (*p* < 0.001) conditions (Figure 3A). The TS-only group exhibited statistically similar rates of antisaccade errors (*p* = 1.0) in the Gap800 and Gap200 conditions and both were significantly higher than those in the Overlap200 condition (*P*-values <0.01). In the TS+ADHD group, antisaccade errors were the highest in the Gap200 condition, which was significantly higher than those observed in the Gap800 condition (*p* = 0.01). The TS+ADHD+OCD group's antisaccade error rates did not alter across the three conditions (all *p*-values = 1.0) (Figure 3A). For more details see Table 1.

#### Group differences

The TS+ADHD group displayed significantly higher antisaccade error rates relative to those of the TS-only group (*p* = 0.02) in the Overlap200 condition. The TS+ADHD+OCD displayed significantly lower rates of antisaccade errors relative to those of the Control group (*p* < 0.001) and to those of the TS+ADHD (*p* < 0.001) in the Gap200 condition. These group differences in the Gap200 condition have been discussed in another report (Tajik-Parvinchi and Sandor, 2012).

### Enhanced ability in TS+ADHD+OCD?

The above results appear to suggest an enhanced antisaccade ability in the TS+ADHD+OCD group. However, given that this group was the oldest group, it is possible that they performed better due to their older age. In order to explore this possibility, two analyses were carried out. First, the main effect of Age and all possible interactions were examined. Two ANOVAs were run, one for “Antisaccade Latency” and one for “Antisaccade Error.” These two variables were selected given the significant group differences that were revealed here. In both analyses, the factor “Age,” “Group,” and “Condition” were entered as the between subjects factors. In antisaccade latency, there were no significant effects of Age *F*_(7, 11)_ = 0.510, *p* = 0.809; Group^*^Age, *F*_(10, 11)_ = 1.176, *p* = 0.395; Condition^*^Age, (14, 22) = 1.081, *p* = 0.423; and Group^*^Condition^*^Age, *F*_(18, 22)_ = 0.994, *p* = 0.499. In the antisaccade error rate, the main effect of “Age” was not significant *F*_(7, 11)_ = 1.688. Similarly, there were no significant effects of Group^*^Age *F*_(10, 11)_ = 0.430, *p* = 0.903; Condition^*^Age *F*_(14, 22)_ = 0.751, *p* = 0.706 or Group^*^Condition^*^Age *F*_(18, 22)_ = 0.638, *p* = 0.832.

In order to further explore the contribution of “Age” to the enhanced saccadic ability displayed by the TS+ADHD+OCD group, each group was age-adjusted by the means of data exclusion of the youngest(s) or the oldest(s) participant(s) in order to close the gap between the average ages of the groups. The data of the two youngest participants in the Control and the TS-only groups, the four youngest participants in the TS+ADHD and the oldest participant in the TS+ADHD+OCD group were excluded (Age-adjusted Control *M* = 168 m, *SD* = 22.38, *n* = 8; TS-only: 143.8 m, *SD* = 11.2, *n* = 4; TS+ADHD: *M* = 164.6, *n* = 5, *SD* = 22.8, TS+ADHD+OCD: *M* = 169 m, *SD* = 18.9, *n* = 6). The above analyses were repeated on the age-adjusted groups.

Despite the loss in power, the overall pattern of findings remained the same. The ANOVA after the age-adjusted groups for antisaccade latency still revealed a significant main effect of Condition *F*_(2, 47)_ = 42.908, *p* < 0.001 and a significant interaction effect *F*_(6, 47)_ = 2.538, *p* = 0.033. The TS+ADHD+OCD group still exhibited significantly shorter antisaccade latencies relative to those of the Control (*p* = 0.004), the TS-only (*p* = 0.006) and the TS+ADHD (*P* < 0.001) groups in the Overlap200 condition (Figure 2B). In antisaccade error rate, a significant main effect of Condition *F*_(2, 47)_ = 3.189, *p* = 0.05 was still present. However, the Group × Condition interaction effect was no longer significant *F*_(6, 47)_ = 1.245, *p* = 0.3. Nevertheless, the TS+ADHD+OCD group had significantly lower antisaccade errors relative to the Control group *p* = 0.001 and to the TS+ADHD group *p* = 0.03 in the Gap200 condition, but their antisaccade error was no longer significantly reduced relative to the TS-only group *p* = 0.06 (Figure 3B). The disappearance of this latter effect may be attributed to a decrease in power as a result of data exclusion. Nevertheless, the overall pattern of enhanced antisaccade ability was still observed in the TS+ADHD+OCD group.

In order to determine whether a significant difference in ADHD symptom severity between the TS+ADHD+OCD and the TS+ADHD groups was contributing to the group differences, an ANOVA was carried out to examine the differences between CGI-at-time and CGI-life-time between these two groups. The CGI at time of data collection *F*_(1, 14)_ = 0.847, *p* = 0.373 and the CGI-life-time *F*_(1, 14)_ = 0.011, *p* = 0.918 were not significantly different between the TS+ADHD and the TS+ADHD+OCD groups. Thus, taken together, these results indicate that ADHD symptom severity and the factor “Age” did not play a key role in the enhanced pattern of performance observed in the TS+ADHD+OCD group.

### Tic severity

Univariate analyses of variances were carried out with Tic Severity as the independent variable and Antisaccade Error rate, Antisaccade latency and Prosaccade Latency as the dependent variables. These analyses did not reveal any significant effects of tic severity.

## Discussion

The results of the present study have revealed several main observations. The Gap-effect was observed in both prosaccades and antisaccades of all of our groups consistent with earlier reports (Munoz et al., 1998; Klein and Foerster, 2001). The Gap-effect Reversal was observed in prosaccades. This finding is consistent with those of the earlier reports in healthy adults (Fischer and Weber, 1997; Knox, 2009). However, in the antisaccade tasks, the results were less uniform as each group displayed unique antisaccade behavior under different conditions and the Gap-effect Reversal was not observed.

There were several important group findings. First, the TS+ADHD+OCD group displayed an enhanced antisaccade ability, reflected by their shorter antisaccade latencies and lower antisaccade error rates. Second, their antisaccade latencies and error rates remained relatively unchanged under various Gap and Overlap conditions. Finally, the TS+ADHD group displayed an overall pattern of longer saccadic latency. This was reflected by their significantly longer latencies in three conditions (prosaccade Gap800, prosaccade Overlap200 and antisaccade Gap800) relative to those of the Control group and in two conditions (antisaccade Gap800 and antisaccade Overlap200) relative to those of the TS+ADHD+OCD group. These findings and their implications will be discussed in the following paragraphs.

### Gap-effect

The latency difference between Gap and Overlap conditions were greater for antisaccades than for prosaccades (Table 1). This observation is in contrast to those reported by Klein and Foerster (2001) who found a somewhat stronger Gap/Overlap difference in prosaccades than antisaccades. One difference between the present study and that study is that our Overlap condition consisted of an overlap of 200 ms whereas in the Klein and Foerster study the overlap conditions consisted of overlap durations of about1000 ms. It is difficult to parse out which proportion of this difference can be attributed to introduction of a Gap condition and which to the introduction of a shorter Overlap condition.

### Gap-effect reversal

#### Prosaccade latency

**Figure 1:**
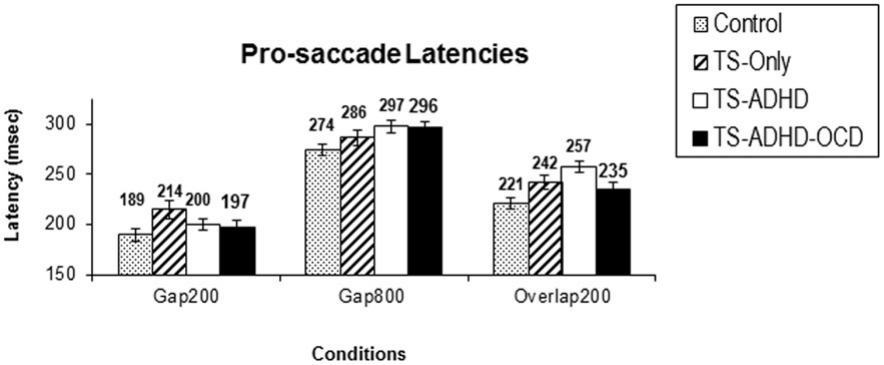
**Prosaccade latencies**. The top graph **(A)** depicts the mean prosaccade latencies of the groups: Control, Tourette-only (TS-only), Tourette Syndrome + Attention Deficit Hyperactivity Disorder (TS+ADHD) and Tourette Syndrome + Attention Deficit Hyperactivity Disorder + Obsessive Compulsive Disorder (TS+ADHD+OCD). The bottom chart **(B)** shows the age-adjusted prosaccade latency of the same groups **(B)**. The top graph **(A)** demonstrates the expected observation of longer saccadic latencies in a longer Gap condition (Gap800) relative an Overlap (Overlap200) condition. This pattern remains the same after each group is age-adjusted **(B)**. Error bars indicate standard errors.

All groups displayed the Gap-effect Reversal (Figure 1A) for prosaccades. The present study is the first to demonstrate the Gap-effect Reversal in prosaccades of healthy children and children with TS. This is consistent with earlier reports in healthy adults and in primates (Dorris and Munoz, 1995; Fischer and Weber, 1997; Knox, 2009). These findings support the notion that the neural mechanism responsible for prosaccade generation is functionally mature in healthy children older than 8 years of age (Tajik-Parvinchi et al., 2003; Luna et al., 2004) and this neural network appears to be unaffected in children with TS.

#### Antisaccade latency and error

**Figure 2:**
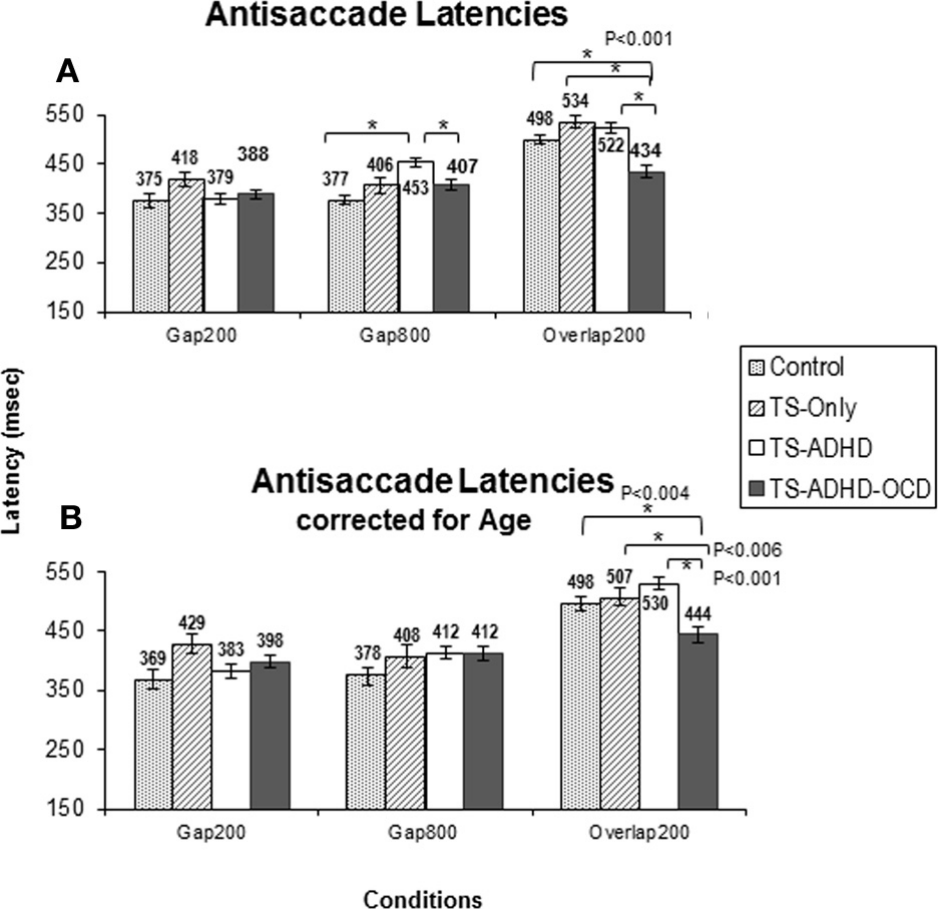
**The graph depicts the mean prosaccade latencies of the groups: Control, Tourette-only (TS-only), Tourette Syndrome + Attention Deficit Hyperactivity Disorder (TS+ADHD) and Tourette Syndrome + Attention Deficit Hyperactivity Disorder + Obsessive Compulsive Disorder (TS+ADHD+OCD)**. It shows the expected observation of longer saccadic latencies in a long Gap condition (Gap800) relative to an Overlap (Overlap200) condition. Error bars indicate standard errors.

The present study is the first to demonstrate that healthy children, children with TS-only and those with TS+ADHD+OCD do not display the Gap-effect Reversal for antisaccades at all. This finding is different from the prosaccade finding described above (Figure 1A) and also different from the earlier reports in healthy adults (Fischer and Weber, 1997). Our TS+ADHD group demonstrated a partial Gap-effect Reversal since their antisaccade latency increased in the Gap800 condition only relative to the Gap200 condition, but not relative to the Overlap200 (Figure 2A). The TS + ADHD + OCD group's antisaccade profile did not vary much between the conditions (Figure 2A). The only antisaccade latency alteration which was observed in this group was a Gap-effect. Our findings from antisaccadic error rates demonstrate similar patterns.

Earlier reports indicate that antisaccade error rates decrease in a long Gap condition (600–800 ms) relative to both a short Gap (200–300 ms) and an Overlap conditions (Fischer and Weber, 1997). In the present study, the healthy Control and the TS+ADHD groups displayed findings somewhat similar to those reported in the healthy adult population. These two groups exhibited a significantly lower number of antisaccade errors in the Gap800 condition only relative to those in the Gap200 condition. In contrast, the TS+ADHD+OCD group exhibited essentially the same unvarying antisaccadic pattern. They displayed relatively low antisaccade error rates across the different fixation offset conditions. These findings support the notion of different neural mechanisms associated with prosaccades and antisaccades (Everling and Munoz, 2000; Malone and Iacono, 2002; Hutton and Ettinger, 2006) and corroborate the earlier reports that the underlying mechanisms associated with antisaccades are still maturing in healthy children 8–16 years of age (Fischer et al., 1997; Munoz et al., 1998; Fukushima et al., 2000; Klein and Foerster, 2001; Eenshuistra et al., 2007). These views are further supported by the observation that the strength of Gap-effect varies between antisaccades and prosaccades.

### Group differences in antisaccade latency error rate

Some of the important findings to note here are the observations that the TS+ADHD+OCD group displayed significantly shorter antisaccade latencies relative to all of the other groups in the Overlap200 condition and significantly shorter antisaccade latencies relative to those of the TS+ADHD group in the Gap800 condition. A speed-accuracy trade-off cannot explain their shorter antisaccade latencies, since the TS+ADHD+OCD group did not display a significant increase in their antisaccade error rates relative to other groups in the same condition. In fact, they exhibited significantly lower rates of antisaccade error in the Gap200 condition, a condition in which an increase in error rates is expected (Fischer et al., 1997; Goldring and Fischer, 1997), suggesting that these children have an enhanced ability to inhibit the GO motor plan when performing antisaccades. Our “Age” analyses indicated that this enhanced performance was independence of an “Age effect.” These observations suggest an enhanced antisaccade ability in children with TS+ADHD+OCD that becomes evident when antisaccade abilities are explored further in such conditions as Gap800 and Overlap200 conditions. A possible explanation may be that the TS+ADHD+OCD group has an enhanced oculomotor ability which allows for ceiling performance regardless of variations in gap/overlap conditions. Thus, the significant performance differences between this group and the other groups may be emerging from variability in the performance of the other groups as a result of Gap/Overlap conditions. In other words, although other groups display costs in performance depending on the gap/overlap condition, the TS+ADHD+OCD group's antisaccade performance remain at an upper limit and relatively unchanged across the conditions. This hypothesis is supported by findings from other studies.

Recent evidence supporting an enhanced motor or cognitive ability in TS has been emerging (Mueller et al., 2006; Jackson et al., 2007, 2011; Tajik-Parvinchi and Sandor, 2011, 2012). Mueller et al. (2006) observed that their sample of children with TS (children with comorbid ADHD were excluded) did not display a “cost” in performance when forced to switch between prosaccades and antisaccades in mixed-blocks of prosaccade and antisaccade (antisaccades and prosaccades were mixed in a block of trials and the color of the fixation dot indicated the type of the eye movement required in the next trial) unlike their healthy children. Jackson et al. (2007) replicated and extended the Mueller et al. (2006) study and observed the same pattern of findings in children with TS without the comorbid ADHD. They concluded that their findings provided evidence that children with TS possess enhanced cognitive control. Jackson et al. (2011) examined this hypothesis of enhanced cognitive control in children with pure-TS (children with comorbid ADHD and OCD were excluded from this sample, but patient participants were receiving medication) in a manual motor control task. They also used Diffusion-Weighted Imaging to investigate the myelination and density of axonal fibers in the prefrontal cortex in patients with TS. They confirmed the hypothesis of an enhanced motor ability in children with TS and further observed reduced fractional anisotropy and increased mean diffusivity in the prefrontal cortex of children with TS relative to those of the control group. They also found that the enhanced motor control in the TS group was predicted by white matter microstructure within the prefrontal cortex. Jackson et al. proposed that children with TS learn to suppress their tics in certain circumstances and thus gain control over their tic symptoms by developing an enhanced control over their motor output. They further proposed that this control leads to changes in the microstructure of the white matter connecting the lateral and medial areas of the prefrontal cortex (forceps minor) and the neural networks connecting the prefrontal cortex with the primary and secondary motor areas leading to a unique neurodevelopmental trajectory in these children.

Our observation of an enhanced saccadic ability in children with TS+ADHD+OCD suggests that an enhanced motor ability may be present not only in pure-TS groups but also in TS+comorbid groups. This adaptive ability may result not only from suppressing tic symptoms but from frequently inhibiting “unwanted behavior” such as tics, hyperactivity, compulsive, and obsessive urges. Earlier reports support the existence of a domain general inhibitory control mechanism which appears to underlie many inhibitory tasks (Friedman and Miyake, 2004). This notion is further supported by functional neuroimaging studies revealing the same neural net-work (the pre-supplementary motor area, the inferior frontal gyrus and the subthalamic nucleus) subserving various inhibitory tasks such as inhibiting eye movements (Chikazoe et al., 2007), suppressing linguistic functions Xue et al. (2008), inhibiting memories, and emotions (Depue et al., 2007). It is possible that the repeated exercise of suppressing various unwanted behavior enhances the domain general inhibitory mechanism producing improved control on any task that demands the resources of this ability. Given that the antisaccade task is a measure of inhibitory control (Hallett, 1978; Munoz and Istvan, 1998; Fukushima et al., 2000), if this hypothesis is correct, performance on the antisaccade task would be improved.

The TS+ADHD+OCD group was the only group who displayed an enhanced antisaccade ability. In line with our suppression practice hypothesis, it is plausible that the TS+ADHD+OCD group exercises inhibitory control in various contexts as a result of controlling a range of unwanted behavior such as unwanted urges, tics, and inattentiveness thus demanding the resources of a domain general inhibitory mechanism more frequently and more intensely. This view has also been used in the rehabilitation field. For example, cognitive training asserts that repeated exercise of an ability of interest over several weeks can lead to enhanced performance on any task that demands that ability (Jaeggi et al., 2008; Klingberg, 2010).

An alternative explanation for the fact that an enhanced antisaccade ability was only observed in the TS+ADHD+OCD group may be that the presence of the comorbid OCD serves as a protective factor. There is some evidence to suggest that genetic variations that may predispose one to certain disorders may protect against other disorders (Rzhetsky et al., 2007). It is also possible that children with a diagnosis of TS+ADHD+OCD present with a unique cognitive profile. This latter notion is supported by the nosological hypothesis (Greimel et al., 2008) which maintains that a combination of tic+ADHD disorders results in a unique behavioral profile not predictable from the knowledge of its individual conditions. Presently, the factors underlying this observation are unclear. Further research is required to examine various hypotheses in different sub-groups of children with TS (e.g., TS+OCD).

### Neural correlates associated with pro/antisaccades and gap/overlap conditions

The neural correlates of these effects have been investigated by single cell recording methods. These studies have demonstrated that the large proportion of the neurons in the FEF increase their activity during the Gap period (their activity level was associated with shorter latencies) relative to the Overlap condition (Everling et al., 1998) and fixation neurons in the SC reduced their activity during this period (Dorris and Munoz, 1995). The frontal cortex has direct inhibitory and excitatory pathways to the SC, in addition to the indirect pathways via caudate nucleus to the SC (Lasker and Zee, 1997). The inverse pattern of activities in the FEF and the SC in the Gap trials implicates the direct pathways from the FEF to the SC as the main area responsible for variations in saccadic latency as a result of the Gap/Overlap manipulations (Everling and Munoz, 2000). Dorris and Munoz (1995) recorded the activity of the fixation cells in the SC of the monkey and found that these neurons decreased their activity for Gap durations of 200–300 ms, but increased their activity for longer Gap durations (400, 600, and 800 ms). It is plausible that variations in the functional activity of this network are responsible for the observed differences in antisaccadic profiles of our groups. For example, The lack of alterations in the antisaccade latency and error rates observed in the TS+ADHD+OCD group suggest a lack of variability in the functional activity in this network as a consequence of Gap and Overlap manipulations, possibly because it consistently functions near the maximal inhibitory capacity.

The neural substrates underlying antisaccade generation has been shown to involve the FEF, supplementary eye field, the intraparietal sulcus, the dorsolateral prefrontal cortex and the anterior cingulated cortex (Sweeney et al., 1996; Connolly et al., 2005; Ford et al., 2005). The FEF has been shown to play an important role in the disengagement from central fixation, the control of contralateral saccades, and saccade prediction (Rivaud et al., 1994). Connolly et al. (2002) demonstrated more intense blood-oxygenation level-dependent activity in the FEF associated with antisaccades. They later replicated and extended their findings (Connolly et al., 2005) to include an observation that pre-target activity during the Gap period in the FEF was inversely correlated with antisaccade latency. This study demonstrated that antisaccades with shorter latencies were associated with higher activities in the early phase of a “build-up” of a response in FEF. However, for prosaccades, this association was only evident in the late phase of the response rise. The investigators interpreted this as an indication that preparatory activity for prosaccades was evident just prior to target presentation and that this difference in “build-up” activity reflected different neural mechanisms associated with prosaccades vs. antisaccades. The findings of the present study support this conclusion. This is reflected by the saccadic alterations in prosaccades, consistent with those reported in healthy adults, but not observed in antisaccades of healthy children.

Reuter et al. (2010) used an event-related functional Magnetic Resonance Imaging (fMRI) study to determine the neural substrates associated with the inhibitory component of the antisaccade task and those associated with the volitional generation of antisaccades. These investigators found a strong association between the volitional generation of an antisaccade and the FEF and the supplementary eye field. They found that the inhibitory component of the antisaccade task was associated with a fronto-parietal network. They did not find a specific network underlying the combined inhibition and volitional generation. They interpreted this finding as evidence suggesting that the neural network associated with the inhibitory and the volitional aspects of the antisaccade task are largely independent. Ettinger et al. (2008) have reported similar findings indicating different neural networks subserving the inhibitory component and the antisaccade generation in antisaccade tasks. The findings of the present study support this hypothesis and further suggest that the neural substrates underlying the inhibitory component of the antisaccade task are maturing faster than those underlying the volitional generation of antisaccades leading to inhibitory performances more similar to those of the healthy adult population. This is reflected by the observation that when a long gap manipulation, Gap800, is applied to the antisaccade task, the healthy children's antisaccade latency does not increase relative to those in the Gap200 and the Overlap200 conditions, unlike those reported in healthy adults. However, the same manipulation reduces their antisaccade error rates significantly in the Gap800 condition relative to the Gap200 condition (similar to those reported in healthy adults), but not relative to the Overlap200 condition (different from those reported in healthy adults). Further research is required to investigate this hypothesis.

### Limitations of the present study

Certain limitations in the present study need to be addressed. Many of our patients were receiving medications at the time of data collection. Requesting the children who were receiving medication to stop for the purposes of the present study was not ethical. Also, during the period open to data collection (2003–2009) the children who were medication-free comprised a very small portion of this population. This raises two important issues. First, the generalizability of data of a TS medication-free group would be highly limited since they appear to make up a small population within this patient group. Second, the medication-free child probably exhibits very mild symptoms which do not require medication intervention. Hence, findings emerging from a medication-free group with TS would have its own limitations.

The results of many oculomotor studies have shown that antipsychotic medications did not alter their pattern of significant findings (Crawford et al., 1995; Georgiou et al., 1995; Lynch et al., 1997; Straube et al., 1997; Green and King, 1998; Levasseur et al., 2001). Furthermore, in a recent paper Reilly et al. (2008) reviewed the effect of different medications on eye movements of healthy and patient participants. The most consistent finding from the reviewed studies on many classes of medications including benzodiazepines, first and second generation antipsychotics and antidepressants, was a decrease in peak saccadic velocity in healthy participants. No significant effects on saccadic latency were reported. In other BG disorders such as Huntington's disease, Reilly et al. reported no effects of haloperidol and sulpiride on antisaccade performance. Since, saccadic peak velocity was not examined in the present study alterations of this parameter as a result of medications is not relevant to the present study.

**Figure 3:**
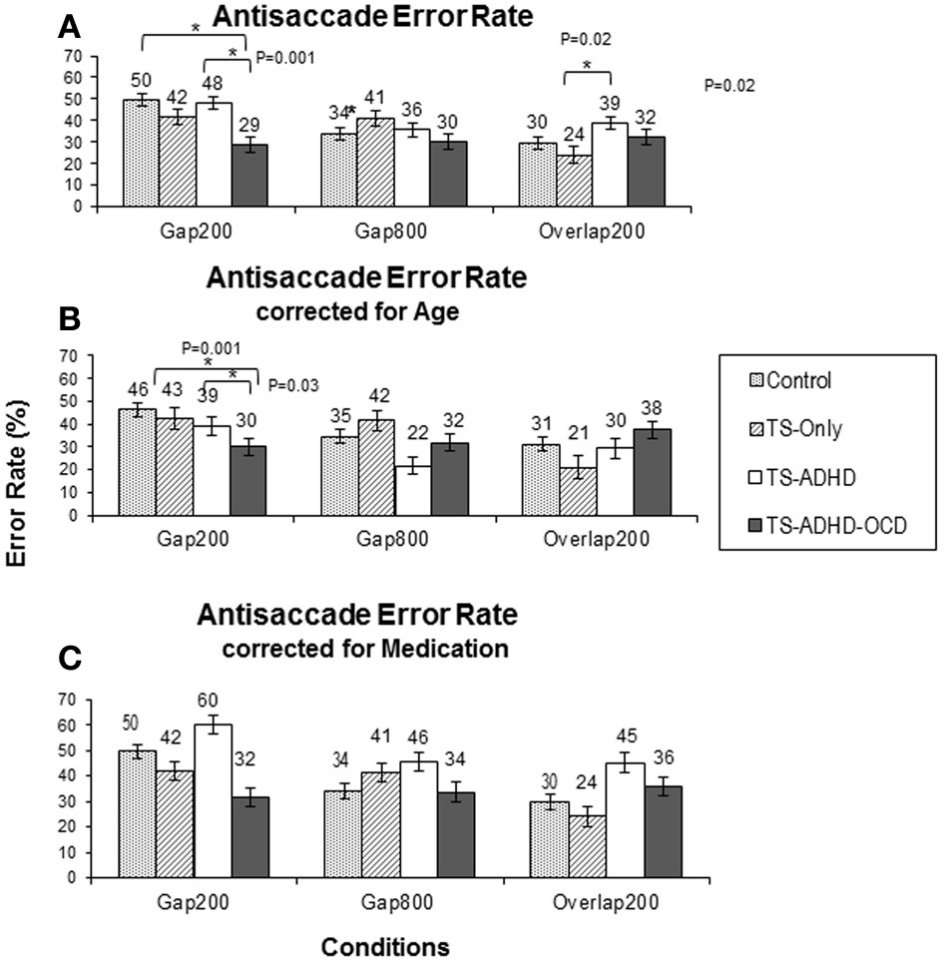
**Antisaccade latency**. The top graph **(A)** shows the mean antisaccade latency of each group: Control, Tourette-only (TS-only), TS + Attention Deficit Hyperactivity Disorder (TS + ADHD) and TS + ADHD+ Obsessive Compulsive Disorder (TS + ADHD + OCD) in each fixation offset condition. The expected longer saccade latency in the Gap800 condition relative to Gap200 and Overlap200 is not observed here. This pattern remains the same following Age-adjustments of each group in the bottom graph **(B)**. The shorter saccadic latency of the TS + ADHD + OCD group relative to the other groups is still present following Age-adjustments in the Overlap200 condition **(B)**. The error bars display standard errors.

A study by Burke and Reveley (2002) indicated an improvement of antisaccade error rate with Risperidone in adult patients with Schizophrenia. In the present study, a number of our participants in the TS+ADHD group (three patients) and in our TS+ADHD+OCD group (two patients) were receiving Risperidone. In order to verify whether the significant effects of antisaccade error rate were the result of this medication, the data of the patients who were receiving Risperidone at the time of data collection were excluded and the analysis regarding antisaccade error rates was repeated. Although following this data exclusion, the average antisaccade error rates of the TS+ADHD and the TS+ADHD+OCD groups increased, the pattern of our findings, including the significant differences, still remained (Figure 3C). Hence, a medication effect does not explain the significant differences in antisaccade error rates between our groups.

Although medication effect on saccadic ability is a valid concern and our readers should exercise caution when interpreting the present findings, a medication effect cannot adequately explain our pattern of findings. This conclusion is supported by the findings of other researchers who have made similar observations and have reported enhanced motor abilities in some of the children with TS (Mueller et al., 2006; Jackson et al., 2007, 2011).

Also, the average ages of our groups were somewhat different. Although the age-adjusted groups exhibited an overall pattern of finding similar to that prior to age-adjustments, the average age of the TS-only group was still not well-matched with the other groups. Hence, the reader should exercise caution when interpreting the findings relevant to this group. While the factor “Age” is a relevant variable to control, in the present study the enhanced performance of the TS+ADHD+OCD group is independent of the factor “Age.”

Lastly, the small size of our samples may have subjected our findings to Type-II errors. Although we cannot exclude the possibility that further significant effects may surface with larger sample sizes, the significant findings which have been detected in the present study were large enough to be captured by our sample sizes and are valid to report.

### Conflict of interest statement

The authors declare that the research was conducted in the absence of any commercial or financial relationships that could be construed as a potential conflict of interest.
